# Assessment of a Size-Based Method for Enriching Circulating Tumour Cells in Colorectal Cancer

**DOI:** 10.3390/cancers14143446

**Published:** 2022-07-15

**Authors:** Sai Shyam Vasantharajan, Edward Barnett, Elin S. Gray, John L. McCall, Euan J. Rodger, Michael R. Eccles, Fran Munro, Sharon Pattison, Aniruddha Chatterjee

**Affiliations:** 1 Department of Pathology, Dunedin School of Medicine, University of Otago, Dunedin 9016, New Zealand; vasai965@student.otago.ac.nz (S.S.V.); bared920@student.otago.ac.nz (E.B.); euan.rodger@otago.ac.nz (E.J.R.); michael.eccles@otago.ac.nz (M.R.E.); 2 Centre for Precision Health and School of Medical and Health Sciences, Edith Cowan University, Joondalup, WA 6027, Australia; e.gray@ecu.edu.au; 3 Department of Surgical Sciences, Dunedin School of Medicine, University of Otago, Dunedin 9054, New Zealand; john.mccall@otago.ac.nz (J.L.M.); fran.munro@otago.ac.nz (F.M.); 4 Department of Medicine, Dunedin School of Medicine, University of Otago, Dunedin 9054, New Zealand; 5 School of Health Sciences and Technology, UPES University, Dehradun 248007, India

**Keywords:** metastasis, colorectal cancer, circulating tumour cell, minimally invasive, biomarker

## Abstract

**Simple Summary:**

Circulating tumour cells (CTC) are metastatic seeds that arise from solid tumours and drive the metastatic spread of malignancy. A better understanding of the biology of CTCs is critical to enable its clinical application for diagnosis and as a therapeutic target. Currently, CellSearch is the only method approved by the U.S. Food and Drug Administration (FDA) for CTC enrichment. However, it isolates only epithelial CTCs and is resource-intensive, which has led to an interest in developing simpler size-based CTC isolation methods. The MetaCell CTC enrichment kit is a size-based method which has been previously used for enriching and culturing CTCs in vitro from several cancer types. Herein, we evaluate the MetaCell platform on its efficacy for retaining colorectal cancer (CRC) cells spiked into blood and the extent of purity of the enriched CTC fraction, which has not been described in previous studies utilising MetaCell. We subsequently applied this method to colorectal cancer patient blood samples and successfully enriched CTCs.

**Abstract:**

Circulating tumour cells (CTC) from solid tumours are a prerequisite for metastasis. Isolating CTCs and understanding their biology is essential for developing new clinical tests and precision oncology. Currently, CellSearch is the only FDA (U.S. Food and Drug Administration)-approved method for CTC enrichment but possesses several drawbacks owing to a reliance on the epithelial cell adhesion molecule (EpCAM) and a resource-intensive nature. Addressing these shortcomings, we optimised an existing size-based method, MetaCell, to enrich CTCs from blood of colorectal cancer (CRC) patients. We evaluated the ability of MetaCell to enrich CTCs by spiking blood with CRC cell lines and assessing the cell recovery rates and WBC depletion via immunostaining and gene expression. We then applied MetaCell to samples from 17 CRC patients and seven controls. Recovery rates were >85% in cell lines, with >95% depletion in WBCs. MetaCell yielded CTCs and CTC clusters in 52.9% and 23.5% of the patients, respectively, without false positives in control patients. CTCs and cluster detection did not correlate with histopathological parameters. Overall, we demonstrated that the MetaCell platform enriched CRC cells with high recovery rates and high purity. Our pilot study also demonstrated the ability of MetaCell to detect CTCs in CRC patients.

## 1. Introduction

Ninety percent of all cancer-related deaths, including those in colorectal cancer (CRC), are attributable to metastasis [1]. There is a strong correlation between diagnosis of colorectal cancer at earlier stages and a better five-year survival rate [2]. The current gold standard for CRC diagnosis is colonoscopy. Colonoscopy is also the most reliable screening test, but its role is limited by high cost, invasiveness, and significant miss rates (15–27%) in detecting small adenomas lesser than 1 cm [3]. Therefore, there is a keen interest in developing a minimally invasive and sensitive means of early detection and therapeutic monitoring of CRC which could be facilitated by the emergence of liquid biopsy techniques. Liquid biopsy involves analysing bodily fluids (usually peripheral venous blood); analytes include circulating tumour DNA (ctDNA), circulating tumour cells (CTCs), and extracellular vesicles [4].

CTCs are tumour cells derived from solid tumours which travel through the blood stream or the lymphatic system [5] and are capable of extravasation and further metastatic dissemination. There has been recent interest in developing CTC-based liquid biopsy technologies for screening, prognostication, and therapeutic monitoring of various cancers, although translation of CTC-based technology for clinical applications remains limited [6,7,8]. This is due to the very low numbers of CTCs found in the blood for most solid cancers, for example, breast cancer (around 6–7 CTCs/7.5 mL blood) [9] and prostate cancer (3–5 CTCs/7.5 mL blood) [9], and the even lower numbers are typically found in colorectal cancer (1–2 CTCs/7.5 mL of blood) [9]. These low numbers pose methodological issues in developing robust methods for CTC enrichment beyond CellSearch [10] for routine isolation of CTCs in clinical settings. Hence, there exists a pressing need to develop reliable methods for CTC enrichment from blood that retain biologically relevant CTC profiles.

In the quest to establish a robust cost-effective technology to routinely enrich CTCs from the whole blood of cancer patients, more than 100 technologies have so far been developed [4,11]. These technologies exploit differences in the biological, electrical, and physical properties of CTCs compared to those of haematopoietic cells, with the aim of enriching CTC populations for downstream analysis or cell culture [12]. The most widely used methods involve enrichment based on combinations of cell surface markers [9,13], each expressed only by either CTCs or leukocytes, allowing easy isolation based on the combination of markers present on each cell. Marker-based methods usually enrich cells expressing the epithelial cell adhesion molecule (EpCAM) and exclude CD45-positive cells, a haematopoietic marker [13,14]. CellSearch is the gold-standard marker-based method and the sole CTC enrichment technology currently approved by the FDA [15]. Previous work with CellSearch has demonstrated that it can routinely enrich CTCs and offer prognostication in many solid cancers, such as breast, colon, and prostate cancers. For example, a study in colorectal cancer patients demonstrated that patients who yielded at least two CTCs had a shorter overall survival (OS) and progression-free survival (PFS) of 10.28 and 5.28 months respectively, compared with those of patients who had ≤1 CTC (OS = 24.84 months; PFS = 8.28 months) [16], highlighting the impact of greater CTC burden, especially after treatment, on the OS and PFS in patients [8,16].

Although there has been much previous research associated with CellSearch and other marker-based methods, EpCAM-based methods have the drawback of enriching only epithelial-like CTCs, resulting in considerable loss of CTCs (~20–40%) [17]. Marker-based methods probably fail to enrich CTCs which have undergone epithelial-to-mesenchymal transition (EMT), a phenomenon that involves the dynamic switching of the phenotype from stromal to mesenchymal resulting in several changes, including the loss of EpCAM expression, which is proposed to aid the survival of CTCs in the bloodstream [1,8,18,19,20]. High-quality CTC enrichment technologies must enrich CTCs along the entirety of the EMT spectrum, which cannot be achieved by marker-based methods such as CellSearch, and reliance on such techniques limits our understanding of CTC biology and metastasis [21]. CTC clusters, often formed by CTCs in intermediate EMT, [22,23] have improved bloodstream survival and metastatic potential when compared to single CTCs, but most current marker-based methods, including CellSearch, fail to capture clusters effectively [24,25,26]. The decreased surface-area-to-volume ratio of cell clusters, in combination with mechanical disruption of clusters in devices with turbulent flow, contributes to the poor efficacy of CellSearch in cluster isolation [22]. Apart from the above-mentioned limitations, CellSearch is also expensive to implement, prohibiting its adoption in smaller laboratories and widespread clinical use. Microfluidic and membrane-based techniques for CTC enrichment have demonstrated the ability to capture CTCs along the EMT axis, isolating a larger spectrum of CTC subpopulations relative to marker-based methods [23]. These advantages expand the repertoire of CTCs for subsequent analysis and provide greater success in the capture of CTC clusters, as demonstrated in particular with a modified ScreenCell method, although further validation with larger sample sizes and a range of cancer types is needed [24].

The MetaCell CTC kit is a size-based CTC enrichment method which has previously been used to enrich and culture CTCs in vitro from a multitude of cancer types, including breast, gynaecological, lung, esophageal, gastric, and urothelial cancers [25,26,27,28,29,30]. To the best of our knowledge, there are no reports evaluating WBC depletion and CTC recovery rates with MetaCell [31], which are important parameters in gauging the performance of CTC enrichment technologies. Herein, we demonstrate the efficacy of the MetaCell kit for CTC enrichment by spiking two different colorectal cancer lines (HCT116 and DLD1) into healthy blood samples and determining recovery rates and WBC depletion rates. We then further apply this method of CTC enrichment to the peripheral blood samples obtained from patients with CRC of various AJCC stages. Finally, we describe future directions for improving the ability of membrane-based methods to capture a wider range of CTC subpopulations and increasing the repertoire of CTCs for subsequent downstream analysis, which could further our understanding of CTC biology and the role of CTCs in metastasis.

## 2. Materials and Methods

### 2.1. Ethical Approval 

Ethical approval was obtained from the University of Otago Human Ethics Committee (H20/003) to obtain blood from healthy volunteers for use in experimental optimisation of the MetaCell workflow for two CRC cell lines. Similarly, a Health and Disability Ethics Committee (HDEC) approval (#14/NTA/33) was obtained in order to collect blood samples from treatment-naïve CRC patients and control patients with nonmalignant bowel disease immediately prior to surgery for characterisation and analysis via MetaCell. All participants provided written informed consent.

### 2.2. Cell Culture 

CRC cell lines (HCT116, DLD1) were obtained from American Type Culture Collection (ATCC), and cell line purity was confirmed by short tandem repeat (STR) profiling. Once thawed, the cells were cultured in Dulbecco’s Minimal Essential Medium (DMEM) supplemented with 10% Fetal Bovine Serum (FBS) and maintained at 37 °C and 5% CO_2_ in a humidified incubator and confirmed to be free of mycoplasma contamination before use in experiments.

### 2.3. Immunostaining to Assess MetaCell Recovery Rates and WBC Depletion Rates

Aliquots of 10, 100, 500, and 10,000 CRC cells (either HCT116 or DLD1) were spiked into four different 8 mL samples of peripheral venous blood drawn from a healthy volunteer into a potassium-EDTA Vacutainer tubes (BD Biosciences). Cells were not added to a fifth blood sample, which served as a negative control. All five blood samples were filtered through MetaCell, and five MetaCell membranes were detached and placed in different wells of a six-well plate. Corresponding aliquots of 10, 100, 500, and 10,000 cells were added directly to four different wells of the six-well plate, serving as an unfiltered control. After eight hours of incubation, the negative control, unfiltered, and MetaCell-filtered cells for the different cell numbers were immunostained [32] (Methods S1 and S2; Tables S1–S3) with CTC (EpCAM and cytokeratins) and WBC markers (CD45 and CD16), and the images for the different cell numbers in the unfiltered and MetaCell-filtered groups were visualised on a Nikon A1+ confocal microscope at 200× magnification. For the unfiltered and the MetaCell-filtered groups containing 10,000 CRC cells, CRC cells retained on the membrane which were positive for either or both CTC markers and negative for the WBC markers were recorded for both groups to qualitatively assess MetaCell’s ability for capturing CRC cells from spiked blood. In groups containing 10, 100, and 500 cells, cells expressing CTC markers and negative for WBC markers were manually counted and recorded for both filtered and unfiltered groups, allowing calculation of recovery rates (Method S2). All of the above experiments were performed using DLD1 and HCT116 cell lines individually.

The depletion of the WBC population was determined by seeding the peripheral mononuclear blood cells (PBMCs) extracted from 8 mL of whole blood from a healthy individual by the density gradient method (Method S2) in a 60 mm culture dish (serving as unfiltered blood), incubated for eight hours at 37 °C, 5% CO_2_, and 95% humidity in RPMI supplemented with 10% FBS. The cells were immunostained with WBC and CTC markers (Method S1; Tables S1–S3) and imaged via confocal microscopy (Nikon A1+ inverted microscope). Similarly, MetaCell-filtered blood without CRC cells (negative control) was stained with WBC and CTC markers. Images of the stained cells were captured for the unfiltered and filtered groups, and mean intensities of Alexa Fluor 647, which was tagged to the WBC markers, was calculated for each of the groups using ImageJ (FIJI). Mean Alexa Fluor 647 intensity was used to calculate the MetaCell WBC depletion rate (Method S2).

### 2.4. Gene Expression Analysis for Assessing Recovery and WBC Depletion Rates for MetaCell

RNA was extracted (RNA Mini Easy kit) from the immunostained cells in unfiltered and the MetaCell-filtered groups. RNA was converted to cDNA using a High-Capacity cDNA reverse transcriptase kit. 1 ng of cDNA was used for a quantitative polymerase chain reaction (qPCR) to measure the expression of the two CTC markers, Cytokeratin 20 (*KRT20*) and *EPCAM*. The expression of both CTC markers was normalised with two housekeeping genes (*RPLP0* and *PPIA*), and the relative fold gene expression was calculated using the 2^−ΔΔCt^ method [33]. The combined gene expression values of the CTC markers in the unfiltered and the MetaCell-filtered groups for the different cell numbers were used to calculate recovery rates (Method S3; Table S4).

For evaluating the WBC depletion rate, RNA from the immunostained cells of the unfiltered blood and negative control (blood without spiked CRC cells) was extracted (RNA Mini Easy kit) and converted to cDNA (High-Capacity cDNA reverse transcriptase kit). 1 ng of cDNA was used for qPCR to measure the expression of the two WBC markers in both groups. The expression of both the WBC markers was normalised with two housekeeping genes (*RPLP0* and *PPIA*), and the relative fold gene expression was calculated using the 2^−ΔΔCt^ method (Method S3). The relative fold gene expression values determined for both groups were used to determine WBC depletion rates (Method S3).

### 2.5. Patient Sampling and Blood Processing 

8 mL peripheral venous blood samples were collected in potassium-EDTA tubes from 17 CRC patients with various stages of cancer (AJCC stages I-IVB) [34,35], and similarly from seven noncancer control patients undergoing surgery for nonmalignant colonic disease (such as Crohn’s disease, ulcerative colitis, and diverticular disease) and were processed within one hour of collection. The characteristics of the CRC patient population in this study are summarised in Table 1. An RBC lysis buffer was added to blood samples (1 mL of blood–5 mL of buffer) and incubated at room temperature for 15 min, after which samples were filtered through MetaCell. Upon completion of the filtration process, the membrane was placed in a six-well plate with 4 mL of RPMI medium supplemented with 10% FBS. The plate was incubated for eight hours at 37 °C, 5% CO_2_, and 95% humidity. These samples were subsequently immunostained in the same manner as the spiked blood samples (Method S1). Based on confocal microscopy, the CTC-positive patients were grouped by whether they had either a single CTC or a CTC cluster (CTC +ve) and patients with a CTC cluster (minimum of two cells attached to each other; cCTC +ve).

### 2.6. Statistical Analysis

All statistical analyses were performed on GraphPad Prism 9 version 9.3.1. For Section 3.2, Section 3.3, Section 3.4 and Section 3.5, the data were represented as the mean ± SD of three biological replicates, for both cell lines in the case of Section 3.2, Section 3.3 and Section 3.4. After checking the data for a normal distribution using the Shapiro–Wilk test, *p*-values were determined by a paired Student’s *t*-test. The Wilcoxon test was used for data without a normal distribution (nonparametric). *p*-values < 0.05 were considered statistically significant. The pathological characteristics were expressed as descriptive statistics. The Fischer exact test was used to determine the association between pathological characteristics and patients identified to have CTCs and clusters. To check for correlation between CEA levels and the number of single CTCs, the *p*-values were determined by Spearman’s rank correlation.

## 3. Results

### 3.1. Qualitative Analysis Demonstrates MetaCell’s Ability for Capturing CRC Cells from Blood Spiked with High CRC Cell Numbers

**Figure 1:**
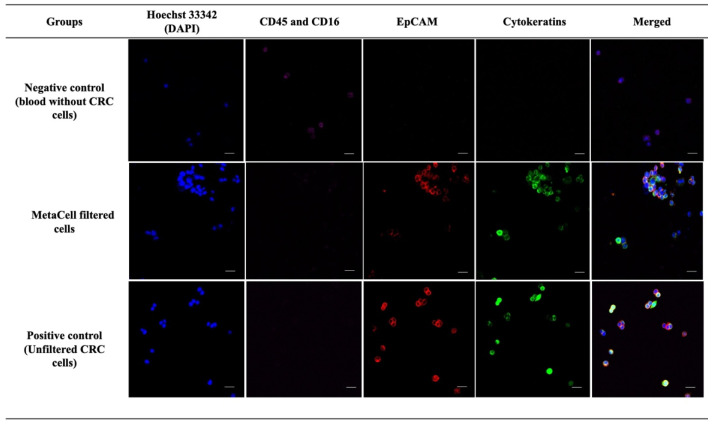
Qualitative assessment of MetaCell’s ability to retain CRC cells spiked into blood. After filtration, the cells retained on the MetaCell membrane from the following samples were stained with Hoechst 33,342 (nuclear stain), white blood cell (WBC) markers (CD45 and CD16), and CTC markers (EpCAM and cytokeratins): unspiked healthy blood (negative control, top row), healthy blood spiked with CRC cells (middle row), and unfiltered CRC cells (positive control, bottom row). The representative images show that unspiked healthy blood cells retained after filtering were positive for WBC markers and lacking CTC markers. Both healthy blood spiked with CRC cells retained on the MetaCell membrane and unfiltered CRC cells were positive for Hoechst 33,342 and CTC markers but negative for WBC markers. Cells were visualised on a Nikon A1+ confocal microscope at 200× magnification. Scale bar represents 80 µm.

We initially qualitatively demonstrated the ability of MetaCell to retain CRC cells from blood spiked with high cell numbers (~10,000 of either HCT116 or DLD CRC cells) by immunostaining. For both HCT116 and DLD1 experiments, cells retained by MetaCell were positive for either or both of the CTC markers and negative for both WBC markers (CD45 and CD16), similar to the unfiltered positive control (Figure 1). In contrast, unspiked blood filtered through MetaCell did not demonstrate any cells positive for CTC markers, only cells positive for WBC markers.

### 3.2. Gene Expression Analysis Confirmed MetaCells’s Ability to Highly Enrich for CRC Cells

**Figure 2:**
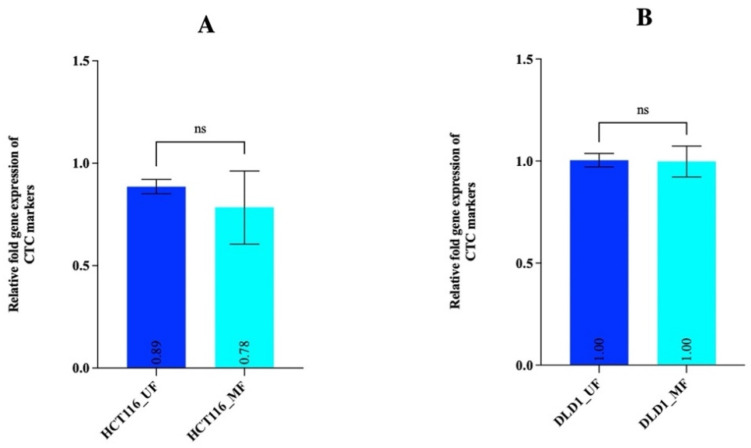
Quantitative analysis of CTC markers in unfiltered and MetaCell-filtered CRC cells. (**A**) Expression of CTC markers (*EPCAM* and *KRT20*) for unfiltered (HCT116_UF) and MetaCell-filtered HCT116 cells (HCT116_MF). (**B**) Expression of CTC markers for unfiltered DLD1 cells (DLD1_UF) and MetaCell-filtered DLD1 cells (DLD1_MF). Error bars represent the mean ± SD of three biological replicates. *p*-values determined by paired Student’s *t*-test; ns *p* > 0.05.

The immunostaining method in Section 3.1 could not be used to determine recovery rates post-filtering by manual counting, as the number of spiked cells was too high (10,000 cells); hence, we used gene expression analysis of two CTC markers (*EPCAM* and *KRT20*) to estimate recovery rates [36]. The relative fold gene expression of CTC markers for each cell line was 0.89 (unfiltered) and 0.78 (filtered) for HCT116, and 1 for both filtered and unfiltered DLD1 cells (Figure 2A,B). The relative fold gene expression values between unfiltered and MetaCell-filtered groups were used to calculate recovery rates and found to be 87.6% (HCT116) and 100% (DLD1 cells).

### 3.3. Quantitative Assessment Demonstrates That MetaCell Recovery Rates Were Not Affected by Low Cell Numbers 

**Figure 3:**
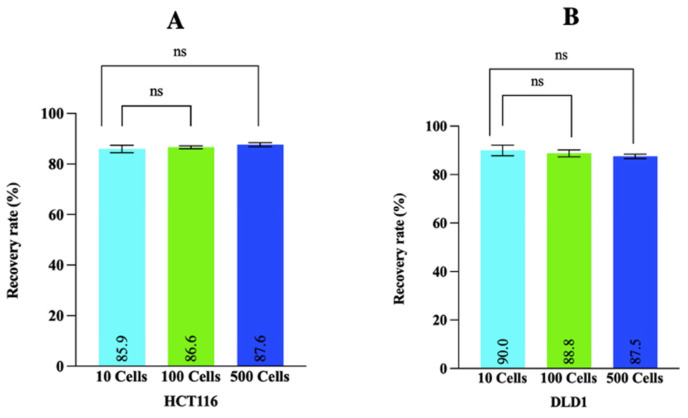
MetaCell recovery rates for healthy blood spiked with different concentrations of CRC cells. Different numbers (10, 100, and 500) of either HCT116 cells (**A**) or DLD1 cells (**B**) were spiked into healthy blood samples and filtered with MetaCell. The cells retained on the membrane and the same number of unfiltered cells were immunostained with CTC (EpCAM or cytokeratins) and WBC markers (CD45 and CD16). Cells that stained positively for Hoechst 33,342 and and at least one of the CTC markers but did not stain for WBC markers were manually counted using a Nikon A1+ confocal microscope at 200× magnification. Recovery rates are represented as the mean ± SD of three biological replicates. *p*-values were determined by a paired *t*-test. ns *p* > 0.05.

The high recovery rates obtained for the MetaCell kit in HCT116 and DLD1 cells (Section 3.2) with high cell numbers (~10,000) prompted the investigation of recovery rates using lower CRC cell numbers (10, 100, 500 cells), which is more reflective of the number of CTCs likely to be present in the circulation of CRC patients. The CRC cells from the unfiltered and MetaCell-filtered groups for the different CRC cell numbers were stained with CTC and WBC markers [37]; cells staining positively for CTC markers and negative for WBC markers were manually counted. For HCT116 cells spiked in blood at 10, 100, and 500, the MetaCell recovery rates were 85.9%, 86.6%, and 87.6%, respectively (Figure 3A). Similarly, for DLD1 cells, the MetaCell recovery rates were 90% (10 cells), 88.8% (100 cells), and 87.5% (500 cells) (Figure 3B). There were no statistically significant differences in MetaCell recovery rates across cell numbers for either cell line assessed in this experiment.

### 3.4. Gene Expression Analysis Revealed Different CRC Cell Numbers Did Not Have a Significant Effect on MetaCell Recovery Rates

**Figure 4:**
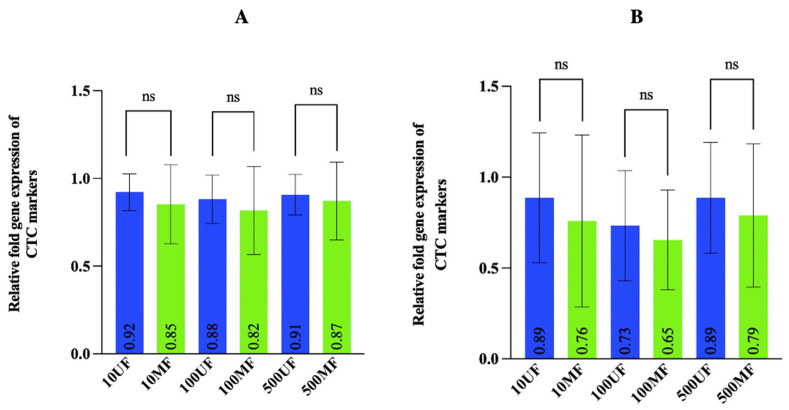
Gene expression analysis to evaluate the effect of cell numbers on the recovery rates for MetaCell. The gene expression analysis of CTC markers (*EpCAM* and *KRT20*) for the different numbers of unfiltered (10, 100, and 500 UF) and MetaCell-filtered HCT116 (**A**) or DLD1 (**B**) cells (10, 100, and 500 MF). Error bars represent the mean ± SD of three biological replicates. *p*-values were determined by paired *t*-tests. ns *p* > 0.05 for both cell lines.

To verify the recovery rates of MetaCell obtained for lower cell numbers (Figure 3A,B) by immunostaining, the recovery rates were secondarily determined for HCT116 and DLD1 cells by gene expression analysis of CTC markers (*EPCAM* and *KRT20*) in the 10, 100, and 500 cell MetaCell-filtered (10, 100, and 500 MF) and unfiltered groups (10, 100, and 500 UF). The average of the combined relative fold gene expression values of both CTC markers in unfiltered HCT116 cells was 0.92 (10 cells), 0.88 (100 cells), and 0.91 (500 cells) and 0.85 (10 cells), 0.82 (100 cells), and 0.87 (500 cells) for the MetaCell-filtered HCT116 cells (Figure 4A). For the DLD1 cells, it was found to be 0.89 (10 cells), 0.73 (100 cells), and 0.89 (500 cells) for the unfiltered group and 0.76 (10 cells), 0.65 (100 cells), and 0.79 (500 cells) for the MetaCell-filtered group (Figure 4B). There was no significant difference in the relative fold gene expression values between the unfiltered and MetaCell-filtered groups for the different cell numbers in both cell lines. The average relative fold gene expression values for CTC markers in unfiltered and the MetaCell-filtered groups was used to calculate recovery rates and were 92.3% (10 cells), 93.1% (100 cells), and 95.6% (500 cells) for HCT116 cells. Similarly, MetaCell recovery rates for DLD1 cells were 85.3% (10 cells), 89% (100 cells), and 88.7% (500 cells).

### 3.5. Estimating WBC Depletion Rate Revealed That MetaCell Significantly Depleted the WBC Population

**Figure 5:**
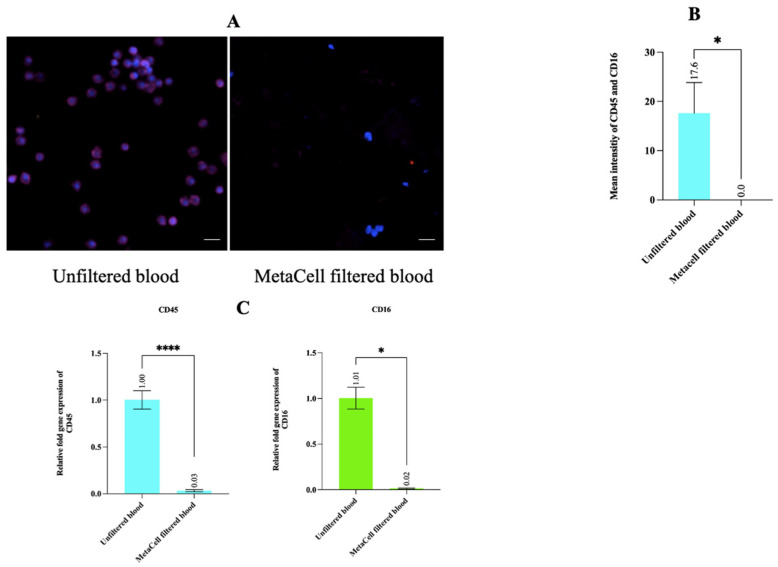
Assessment of WBC depletion in MetaCell-filtered blood by immunostaining and qPCR. (**A**) Representative images of the cells positive for WBC markers (CD45 and CD16) in the unfiltered and MetaCell-filtered blood. The cells positive for CD45 and CD16 are the cells that have a blue-stained nucleus (Hoechst 33,342) with a purple border. The images were taken on a Nikon A1+ confocal microscope at 200× magnification. Scale bar represents 80 µm. (**B**) The mean intensities of the Alexa Fluor 647 conjugated with WBC markers were measured in the unfiltered and the MetaCell-filtered blood. The data is represented as the mean ± SD of three biological replicates with six different fields considered for each group. *p*-values were determined by Wilcoxon matched-pairs test of 3 biological replicates. (**C**) The gene expression analysis of the WBC markers for the filtered and unfiltered blood, presented as the mean ± SD of three biological replicates. Statistical analysis between filtered and unfiltered groups was performed by a paired *t*-test for CD45 and the Wilcoxon test for CD16. (* *p* < 0.05, **** *p* < 0.0001).

In order to assess the depletion of WBCs in the MetaCell-filtered fraction, the images of cells positive for WBC markers (CD45 and CD16) post-immunostaining were recorded by confocal microscopy for MetaCell-filtered blood and unfiltered WBCs (Figure 5A); average mean intensities of the WBC markers in the unfiltered and the MetaCell-filtered blood were 17.6 and zero, respectively (Figure 5B). The difference between intensities of CD45 and CD16 markers between the unfiltered and MetaCell-filtered fraction was statistically significant (*p* = 0.03).

To confirm the depletion in WBCs for the MetaCell-filtered fraction observed by the immunostaining method, the average relative fold gene expression values of the WBC markers were determined by qPCR. Relative fold gene expression values for *CD45* in the two groups were 1.0 (unfiltered) and 0.03 (MetaCell-filtered) (Figure 5C). Similarly, for *CD16*, the relative fold gene expression values were 1.01 (unfiltered) and 0.02 (MetaCell-filtered) (Figure 5C). The gene expression values for *CD45* and *CD16* in the unfiltered group were significantly greater compared with those in the filtered group (*p* < 0.0001 and *p* = 0.03 for *CD45* and *CD16*, respectively). Using these values, WBC depletion in the MetaCell-filtered fraction was 97% (*CD45*) and 98% (*CD16*).

### 3.6. Patient Characteristics and CTC Detection

**Figure 6:**
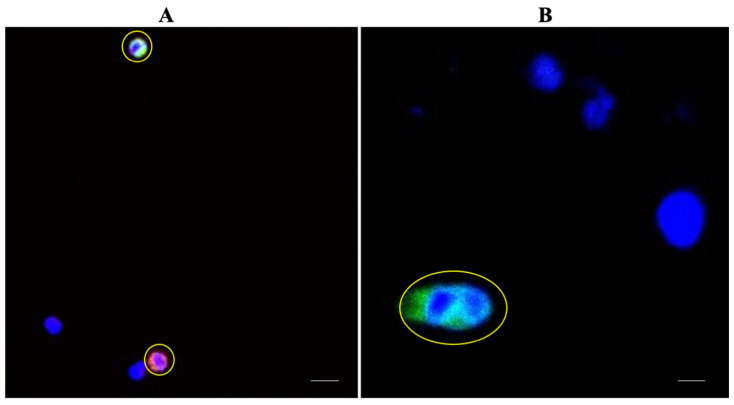
Representative images of single CTCs and a CTC cluster enriched from a CRC patient’s blood. (**A**) Image of two single CTCs (highlighted in yellow) detected in a CRC patient’s blood where one CTC was positive for cytokeratins (green) and the other CTC was positive for EpCAM and cytokeratins (pink), and both were positive for Hoechst 33,342 and negative for WBC markers (CD45 and CD16) when viewed under a Nikon A1+ confocal microscope at 200× magnification. Scale bar represents 80 µm. (**B**) Image of a CTC cluster (highlighted in yellow) detected in a CRC patient’s blood positive for cytokeratins and Hoechst 33,342 when viewed under a Nikon A1+ confocal microscope at 200× magnification. Scale bar represents 80 µm.

We applied MetaCell to enrich CTCs from 17 treatment-naive CRC patients across a range of AJCC stages I–IVB (Table 1) and seven patients with nonmalignant colonic disease. The median age of the patients was 64 (ranging from 43 to 84 years). Applying a cut-off of ≥1 CTC or 1 CTC cluster, we detected either a single CTC (Figure 6A) or a cluster (Figure 6B) in nine out of 17 patients (52.9%), and four out of the nine CTC-positive patients presented with CTC clusters (23.5%). Cells expressing the CTC markers were not observed in patients with nonmalignant colonic disease (Figure S1). Two of these nine CTC-positive patients showed presence of both a single CTC and a cluster. All CTCs we detected were positive for cytokeratins; some also expressed EpCAM, but none expressed EpCAM alone (Table S5).

We assessed the correlation between CTC detection and demographic factors such as age and gender or clinicopathological parameters: AJCC stage, tumour site (left or right colon), or the presence of distant metastasis. We did not observe any significant dependency on AJCC stage, tumour site, or the presence of distant metastasis on the detection of CTC +ve and cCTC +ve patients (Table 2).

As part of normal pathological review, resected tumours were assessed for the presence of normal mismatch repair (MMR) proteins by immunohistochemistry (IHC) and described as MMR proficient (presence of all four MMR proteins: MLH1, PMS2, MSH2, MSH6) or MMR deficient (absence of one or more MMR proteins). We did not observe a significant correlation between detection of CTC +ve or cCTC +ve and the presence or absence of normal MMR proteins in the tumours (Table 2). However, higher percentages of CTC +ve (63.6%) and cCTC +ve patients (27.2%) were seen in patients with proficient MMR. BRAF V600E status was similarly assessed in some resected tumour specimens via IHC. Patients were classified as BRAF V600E positive (presence of mutant protein), BRAF V600E negative (absence of mutant protein), and ND (patients where the status of the BRAF V600E protein was not known). A higher proportion of cCTC+ve (40%) patients was observed for the BRAF V600E-negative group (40%) when compared to that for the BRAF V600E-positive group (14%), although this correlation did not reach statistical significance (Table 2).

**Figure 7:**
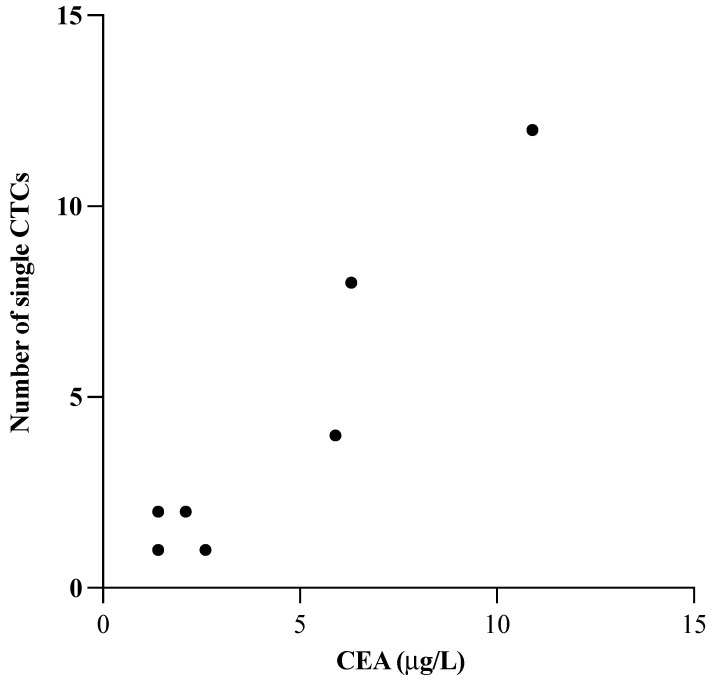
Association of carcinoembryonic antigen (CEA) level (µg/L) and the number of detected single CTCs in CTC-positive patients. *p*-values were determined by Pearson correlation.

Carcinoembryonic antigen (CEA) levels in the blood are commonly measured for CRC prognostication. We noted that in CTC-positive patients, CTC counts were higher with increasing carcinoembryonic antigen (CEA) levels; in patients with CEA levels in the range of 1–3 μg/L, we found 1–2 CTCs, with higher CTC numbers at CEA levels ≥5 μg/L (Figure 7, Table S6). A strong correlation (*p* = 0.008) was seen between the number of single CTCs and the CEA concentrations (Figure 7), although this trend was lost when considering both CTC-positive and negative patients (*p* = 0.645) (Figure S2).

## 4. Discussion

In our study, we used an existing membrane-based technology, MetaCell, for enriching CTCs from CRC patients’ blood. While MetaCell has been previously used for enrichment and in vitro culture of CTCs from various cancer types, there is a lack of studies assessing WBC depletion and recovery rates, which are important parameters for gauging the efficiency of CTC enrichment technologies. Therefore, in this study, we first evaluated these parameters before applying MetaCell for CTC enrichment from CRC patients’ blood. Initially, we demonstrated high recovery rates for MetaCell from healthy blood spiked with high CRC cell numbers (HCT116 and DLD1). We further explored the effect of lower CRC cell numbers (10, 100, and 500 cells) on recovery rates, as the cell concentrations we used initially did not accurately mirror CTC concentrations that circulate in a patient’s blood (1–10 CTCs per mL [38]). We did not observe any difference in recovery rates across different cell concentrations or cell lines; all groups exhibited good recovery rates of at least 85%, using both manual counting and gene expression analysis. These recovery rates are similar to those of CellSearch, estimated to capture around 82% of CTCs, and another microfluidic technology with a similar recovery rate of 85% [39,40]. It would have been ideal to evaluate the performance of MetaCell in blood spiked with <10 CRC cells, which would be more representative of CTC numbers obtained from the blood of CRC patients. However, since we used serial dilution for obtaining cell fractions, increasing the number of dilutions and decreasing the volume of spiked cells would increase error and decrease the likelihood that our spiked cell fraction accurately represents the number of cells we aim to spike. This could have a significant influence on the accuracy of recovery rates obtained for MetaCell at these low cell numbers (<10 CRC cells), hence further dilutions were not undertaken. Additionally, the main reason for performing cell line experiments was to provide evidence of MetaCell’s ability to isolate CTCs, which we considered adequately demonstrated with 10 spiked cells to progress to patient samples.

A consistent drawback of current CTC enrichment methods is the high background of WBCs that are retained in the enriched CTC fraction, which not only provides challenges in the downstream bulk DNA/RNA processing of CTCs but may also disrupt CTC enrichment by clogging the pores of the membrane [41]. We demonstrated that MetaCell was able to deplete >95% of the WBC population via immunostaining and gene expression analysis of the WBC markers, which was similar to the depletion rates achieved by a spiral microfluidic device where over 99% of depletion in WBCs was reported [40]. Our gene expression analysis showed that traces of WBCs remained in the MetaCell-enriched fraction missed by the immunostaining approach, demonstrating that qPCR methods could be a more accurate approach for determining WBC depletion achieved by MetaCell.

The results from our optimisation experiments supported the application of this method for the isolation of CTCs from peripheral blood of 17 CRC patients, presenting at different stages of malignancy, as well as seven patients with nonmalignant colorectal disease. We detected CTCs in 52.9% of CRC patients using MetaCell, which was in concordance with another study utilising a similar filter-based device, isolation by size of tumour cells (ISET), where CTCs were obtained from 52.8% of the CRC patients [42]. The similar proportion of CRC patients without detectable CTCs suggest that a pore size of 8 µm may not be ideal; indeed, it has been previously demonstrated that CTCs in CRC are sometimes smaller than 8 µm [43,44]. The loss of smaller CTCs through the pores of the membrane may explain why nearly half of our cohort did not yield a single CTC. It would be interesting to apply a sequential filtration technique with membranes of different pore sizes (<8 µm) and investigate whether this improves the performance of membrane-based technologies. Capturing not only smaller CTC populations but also single CTCs and clusters from the same patient has been demonstrated in another membrane-based approach for CTC enrichment [24]. Such an approach enables comparative studies on paired single CTCs and CTC clusters to gain a better understanding on the role of these CTC subpopulations in CRC metastasis.

While membrane-based methods such as MetaCell have several advantages, phenotypic characterisation of CTCs enriched by membrane-based methods are usually performed via immunocytochemistry and fluorescence microscopy with only one or two additional markers [45]. This limited our use of markers and prompted us to choose the most popular markers used for CTC characterisation, i.e., EpCAM and cytokeratins [46,47]. Furthermore, using these markers allows some comparison of the efficiency of MetaCell to isolate phenotypically similar cells to that of CellSearch. Although these epithelial markers may be useful in separating WBCs from candidate CTCs, the isolated cells may not in all cases be malignant; previous evidence demonstrates that circulating cells with epithelial markers are present in the blood of patients with nonmalignant colorectal disease, although we observed no such cells in our study [48]. Our dependence upon epithelial markers additionally meant that we could not verify whether MetaCell was able to detect CTCs along the EMT continuum with a loss of these markers, although these CTC populations may be more relevant to metastasis [49]. Further approaches should assess the suitability of a wider range of CTC markers, including mesenchymal and epithelial markers, to investigate if the use of mesenchymal markers could improve the efficiency of MetaCell for enriching CTCs, as this represents a domain in which MetaCell may eclipse the utility of CellSearch.

We also explored the association between the detection of CTCs with several clinicopathological and demographic features. We did not observe any significant correlation between CTC detection and age, gender, AJCC stage, or distant metastasis. Distinct metastatic patterns in colorectal cancer patients exist based on primary tumour location, which prompted us to investigate whether the location of the primary tumour had a bearing on CTC detection [50,51]. In our cohort we did not observe a significant influence of the primary tumour location on CTC detection.

As per clinical guidelines, the assessment of MMR proficiency and the *BRAF* V600E protein in resected tumours is recommended to inform adjuvant treatment selection [52,53]. Impairments in the DNA mismatch repair (MMR) system are caused by a mutation in any one of the four genes (*MLH1*, *PMS2*, *MSH2*, and *MSH6*) that constitute the MMR system. MMR-deficient (dMMR) tumours are more vulnerable to microsatellite instability (MSI), observed in 12–17% of sporadic CRCs. In sporadic CRC, MMR deficiency is often accompanied by *BRAF* V600E mutations occurring due to *MLH1* promoter hypermethylation [54]. Although greater CTC burden may occur in tumours with MSI [55], we did not find any significant correlation between MMR or BRAF V600E status and CTC detection in the present study.

Carcinoembryonic antigen (CEA) is a serum marker with prognostic value in early-stage CRC and may indicate recurrence after treatment. It has been previously demonstrated that the combination of CTC number and CEA levels could provide a more accurate prognosis in CRC patients, due to the range of factors normally affecting CEA levels in vivo [56,57]. We observed no significant correlation between CEA levels and CTC numbers in our entire cohort; however, in the CTC-positive patients only, increasing CEA levels had a positive correlation with the number of CTCs detected. No clear conclusion can be drawn on the relationship between serum CEA and CTC numbers based on the results obtained from our cohort, although our observations warrant further investigation of this association.

CTC clusters represent an intriguing CTC population, with profound recent interest due to their apparently increased metastatic potential; dissociation of CTC clusters decreases the rates of metastasis in mice, suggesting a central role for clusters in cancer spread [58]. We detected CTC clusters in 23% of our cohort, similarly to previous studies where clusters were detected in over 20% of CRC patients using membrane-based isolation [24,42]. These observations highlight the utility of membrane-based methods for capturing CTC clusters. We further investigated the correlation between various demographic and histopathological features and CTC cluster isolation, none of which correlated significantly with cluster detection. As the primary focus of this study was to demonstrate MetaCell’s ability to enrich CTCs from blood of colorectal cancer patients, we enrolled a small cohort of patients after diagnosis and prior to any treatment to maximise the chance of CRC CTC isolation. The enrolment after diagnosis and prior to treatment contributed to the short follow-up duration in our cohort which limited our investigation of reliability of this method for early diagnosis and/or monitoring of the disease and prognostic significance. Our future studies would be directed at establishing a relationship between CTCs enriched using MetaCell and progression-free survival/overall survival.

## 5. Conclusions

CTCs have the potential to be developed as a minimally invasive means for early detection and therapeutic monitoring as they can be detected in the blood of cancer patients. However, their clinical translation is limited due to very low numbers of CTCs that can be found in the blood, coupled with a lack of robust methods for isolation. The first step towards developing these cells as biomarkers is to optimise a method to successfully enrich these rare cells with high purity. Currently, CellSearch is the only FDA-approved method, and has the drawback of enriching only for CTCs that express EpCAM. This shortcoming can be overcome by adopting a size-based approach for CTC enrichment. Here, we have demonstrated the efficiency of a size-based method (MetaCell) for enriching CTCs in two CRC cell lines with reasonably high purity. Our results demonstrated the capability of MetaCell to enrich CTCs from the peripheral blood of CRC patients. It would also be interesting to investigate the efficiency of filtration devices with membranes having different pore sizes so that the smaller CTCs which are missed by the current membrane-based methods could be captured. Sequential filtration could be the way forward as it could improve the efficiency of the membrane-based methods and expand the repertoire of CTCs for further downstream analysis, which could further facilitate exploration the role of these rare tumour cells in colorectal cancer metastasis.

## Figures and Tables

**Table 1:** Patient characteristics. Details of CRC patients used for CTC enrichment by a size-based method (*n* = 17).

Characteristics	Number of Patients (%)
**Age (years)**	
40–70	11 (58.8)
71–80	6 (35.2)
**Gender**	
Male	7 (41.2)
Female	10 (58.8)
**Ethnicity**	
New Zealand European	15 (88.2)
Other European	2 (11.8)
**Clinical Stage (AJCC 8th edition)**	
Early Stage (stage I and II)	7 (41.2)
Late Stage (stage III and IV)	10 (58.8)
**Tumour Site**	
Left sided CRC	10 (58.8)
Right sided CRC	7 (41.2)
**Metastasis**	
No distant metastases	11 (64.7)
Distant metastases present	6 (35.3)
**MMR (IHC)**	
MMR proficient	11 (64.7)
MMR deficient	6 (35.3)
**BRAF (IHC)**	
BRAF V600E −ve	5 (29.4)
BRAF V600E +ve	7 (41.2)
ND	5 (29.4)

Abbreviations: IHC—immunohistochemistry, MMR proficient—all four Mismatch Repair (MMR) proteins (MLH1, PMS2, MSH2, MSH6) present at IHC, MMR deficient—loss of one or more MMR proteins determined by IHC, BRAF—proto-oncogene B-Raf and v-Raf murine sarcoma viral oncogene homolog B, BRAF V600E +ve—patients with the mutant BRAF V600E protein determined by IHC, BRAF V600E −ve—patients without the mutant BRAF V600E protein determined by IHC, ND—BRAF V600E status not determined.

**Table 2:** Association of clinical and pathological variables with patients positive for single CTC and CTC clusters.

Clinical or Pathological Variables	*n*	% CTC +ve Patients (*n*)	% CTC −ve Patients (*n*)	*p*-Value	% cCTC +ve Patients (*n*)	% cCTC −ve Patients (*n*)	*p*-Value
**Age (years)**							
40–70	11	54.5 (6)	45.5 (5)	>0.99	18.2 (2)	81.8 (9)	0.58
71–85	6	50 (3)	50 (3)		33.3 (2)	66.7 (4)	
**Gender**							
Male	7	71.4 (5)	28.6 (2)	0.33	42.8 (3)	57.2 (4)	0.25
Female	10	40 (4)	60 (6)		10 (1)	90 (9)	
**Clinical stage**							
Early stage (I & II)	7	71.4 (5)	28.6 (2)	0.33	14.3 (1)	85.7 (6)	0.60
Late stage (III & IV)	10	40 (4)	60 (6)		30 (3)	70 (7)	
**Tumour site**							
Left side	10	60 (6)	40 (4)	0.63	30 (3)	70 (7)	0.60
Right side	7	42.8 (3)	57.2 (4)		14.3 (1)	85.7 (6)	
**Metastasis**							
No distant metastases	11	54.5 (6)	45.5 (5)	>0.99	18.2 (2)	81.8 (9)	0.58
Distant metastases present	6	50 (3)	50 (3)		33.3 (2)	66.7 (4)	
**MMR (IHC)**							
MMR proficient	11	63.6 (7)	36.4 (4)	0.33	27.2 (3)	72.8 (8)	>0.99
MMR deficient	6	33.3 (2)	66.7 (4)		16.6 (1)	83.4 (5)	
**BRAF V600E (IHC)**							
BRAF −ve	5	40 (2)	60 (3)	>0.99	40 (2)	60 (3)	0.52
BRAF +ve	7	42.8 (3)	57.2 (4)		14.3 (1)	85.7 (6)	
ND	5	80 (4)	20 (1)		20 (1)	80 (4)	

Abbreviations: *n*—total number of patients, CTC +ve—patients positive for single CTC or cluster, cCTC +ve—patients positive for clusters only, IHC—immunohistochemistry, MMR proficient—all four Mismatch Repair (MMR) proteins (MLH1, PMS2, MSH2, MSH6) present at IHC, MMR deficient—loss of one or more MMR proteins determined by IHC, BRAF—proto-oncogene B-Raf and v-Raf murine sarcoma viral oncogene homolog B, BRAF V600E +ve—patients with mutant BRAF V600E protein determined by IHC, BRAF V600E −ve—patients without the mutant BRAF V600E protein determined by IHC, ND—BRAF V600E status not determined. *p*-value indicates significance according to the Fischer’s exact test for testing the relation between clinicopathological parameters and CTC detection.

## Data Availability

The data presented in this study are available on request from the corresponding authors.

## Supplementary Materials

The following supporting information can be downloaded at: https://www.mdpi.com/article/10.3390/cancers14143446/s1, Figure S1: Absence of CTCs in patients with nonmalignant colonic disease, Figure S2: Association between carcinoembryonic antigen (CEA) levels and enumeration of single CTCs, Table S1: Details of commercially available antibodies for immunostaining, Table S2: Optimum antibody dilutions for immunostaining protocol, Table S3: Preparation of the antibody cocktail for CTC characterisation by immunostaining, Table S4: Primer sequences of CTC and WBC markers used for gene expression analysis (qPCR), Table S5: Classification of CTCs/CTC clusters based on the CTC markers (EpCAM and cytokeratins), Table S6: Relation between CEA levels and number of CTCs found in CTC-positive and negative patients, Method S1: Immunostaining protocol for colorectal cancer cell lines, PBMCs, and CRC patient blood samples, Method S2: Assessing the recovery rates and WBC depletion rates for MetaCell by immunostaining, Method S3: Gene expression analysis for calculating recovery rates and WBC depletion rates for MetaCell.
