# Supplementary material for: Assessment of a Size-Based Method for Enriching Circulating Tumour Cells in Colorectal Cancer
Source: Cancers (Basel). 2022 Jul 15;14(14):3446. doi: 10.3390/cancers14143446 (PMC9319975; doi:10.3390/cancers14143446)
Supplement: Supplementary file 1 [file cancers-14-03446-s001.zip › cancers-1775537-supplementary.pdf]

Supplementary Materials

Assessment of a Size-Based Method for Enriching Circulating Tumour Cells in Colorectal Cancer

Sai Shyam Vasantharajan, Edward Barnett, Elin S. Gray, John L. McCall, Euan J. Rodger, Michael R. Eccles, Fran Munro, Sharon Pattison and Aniruddha Chatterjee

Table S1. Commercially available antibodies for CTC characterisation by immunostaining.

Clone	Target	Supplier	Cat#	Fluorophore
CK3-6H5	cytokeratin	Miltenyi Biotec	130-080-101	FITC
C11	cytokeratin	Cell Signalling Technology	4523S	Alexa488
AE1/AE3	cytokeratin	Life Technologies	53-9003-82	Alexa488
VU-1D9	EpCAM	Life Technologies	A15782	PE
HI30	CD45	BioLegend	304018	Alexa647
3G8	CD16	BioLegend	302020	Alexa647

Abbreviation: Cat#—catalogue number.

Table S2. Optimum antibody dilutions for immunostaining protocol.

Antibody	Final Dilution
CD45	1:50
CD16	1:50
EpCAM	1:100
C11	1:100
AE1/AE3	1:200

Table S3. Preparation of the antibody cocktail for CTC characterisation by immunostaining.

Primary	×1 (µL)	×2 (µL)
Normal Donkey Serum (NDS)	11	22
Permeabilisation buffer	82.5	165
CD45	2	4
CD16	2	4
C11	1	2
AE1/AE3	0.5	1
EpCAM	1	2
Final volume of antibody cocktail	100	200

Table S4. Primer sequences of CTC and WBC markers used for gene expression analysis (qPCR).

Genes	Type	FP (5′–3′)	RP (5′–3′)	Company
RPLP0	HKG	TCGTCTTTAAACCCTGCTTG	TGTCTGCTCCCACAATGAAAC	IDT
PPIA	HKG	GTGGCGGATTTGATCATTTGG	CAAGACTGAGATGCACAAGTG	IDT
EpCAM	CTC marker	TGTTGCTGGAATTGTTGTGC	CCTATGCATCTCACCCATCTC	DIT
KRT20	CTC marker	TCCAGTCCCATCTCAGCAT	GCGTTCCATGTTACTCCGAAT	IDT
CD45	WBC marker	AAAGCCCAACACCTTCCC	CAACTGAAGGCTGAACTGTCA	IDT
CD16	WBC marker	GCTTCGACTAGATGGTCCCTA	CACACTGTCCTTCTCAAGCA	IDT

Abbreviations: FP—forward primer, RP—reverse primer, HKG—housekeeping genes, IDT—Integrated DNA Technologies (Coralville, IA, United States)

Table S5. Classification of CTCs/CTC clusters based on the CTC markers (EpCAM and cytokeratins).

Patient ID (#)	EpCAM +ve	CK +ve	EpCAM +ve and CK +ve
CRC_CTC_2	Nil	1 SC and 2 CC	Nil
CRC_CTC_4	Nil	1 SC	1 SC
CRC_CTC_10	Nil	1 CC	Nil
CRC_CTC_11	Nil	4 SC	Nil
CRC_CTC_12	Nil	2 SC and 1 CC	Nil
CRC_CTC_13	Nil	1 CC	Nil
CRC_CTC_14	Nil	1 SC	Nil
CRC_CTC_15	Nil	10 SC	2 CC
CRC_CTC_17	Nil	8 SC	Nil

Abbreviations: CK+ve—cytokeratin-positive, SC—single CTC, CC—CTC cluster.

Table S6. Relation between CEA levels on the number of CTCs found in CTC-positive and negative patients.

CTC Status of CRC patients	CRC Patient ID	CEA (ug/L)	No. of Single CTCs
**CTC +ve**	CRC_CTC_2	1.4	1
	CRC_CTC_14	2.6	1
	CRC_CTC_4	1.4	2
	CRC_CTC_12	2.1	2
	CRC_CTC_11	5.9	4
	CRC_CTC_17	6.3	8
	CRC_CTC_15	10.9	12
**CTC −ve**	CRC_CTC_1	60.9	0
	CRC_CTC_3	4.1	0
	CRC_CTC_5	13.8	0
	CRC_CTC_6	14	0
	CRC_CTC_7	1.8	0
	CRC_CTC_8	1.2	0
	CRC_CTC_9	2.5	0
	CRC_CTC_16	13.4	0

Abbreviations: CTC +ve—patients positive for single CTCs, CTC −ve—patients negative for single CTCs.

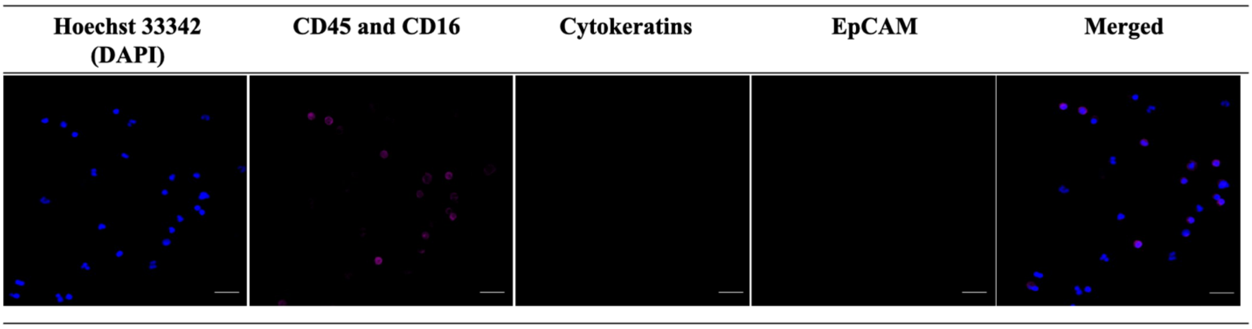

Figure S1. Absence of CTCs in patients with nonmalignant colonic disease. Representative image of cells post-MetaCell filtering of the peripheral blood of patients with nonmalingnant colonic disease, positive for WBC markers and negative for CTC markers when viewed under a Nikon A1+ confocal microscope at 200× magnification. Scale bar represents 36 μm.

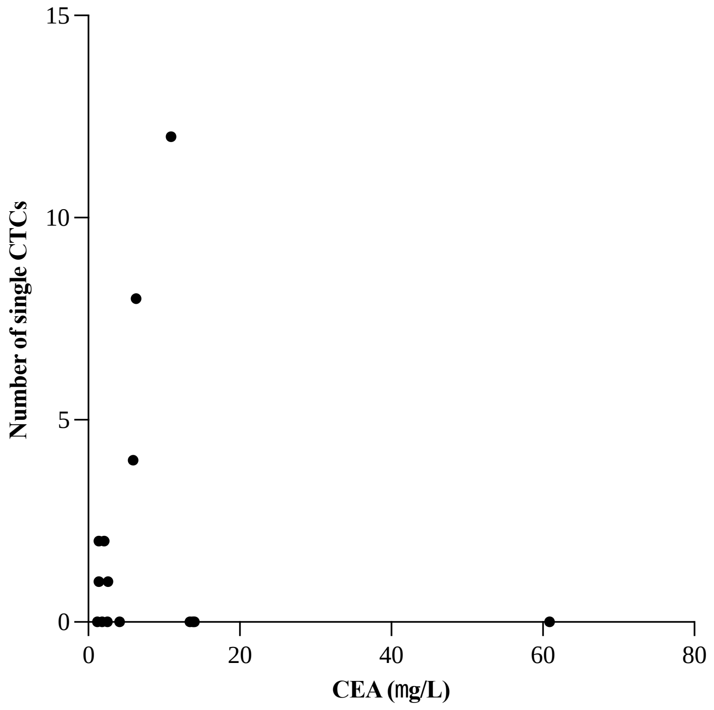

Figure S2. Association between carcinoembryonic antigen (CEA) levels on the enumeration of single CTCs. *p*-values were determined by Spearman's rank correlation.

1. Method S1 Immunostaining protocol for colorectal cancer cell lines, PBMCs, and CRC patient blood samples

After MetaCell filtration and incubation, the cells were washed with 2 mL of PBS and fixed in 200 μL of 4% paraformaldehyde (PFA) for 10 minutes. The cells were then incubated in 200 μL of a permeabilisation buffer (0.2% Triton X-100 in 1× PBS) for 15 minutes. Further, the cells were incubated for 10 minutes in 200 μL of a blocking solution (20 μL of normal donkey serum or filtered foetal bovine serum (FBS) in 180 μL of antibody buffer) to minimise nonspecific binding of antibodies. The cells were then incubated for one hour in an antibody cocktail (Table S1) consisting of cytokeratins, EpCAM, CD45, and CD16 (Tables S2 and S3). After incubation at room temperature for one hour, the cells were washed three times with 1% bovine serum albumin (BSA) in PBS for 5 minutes each, followed by two short PBS washes. One milliliter of PBS with 2 drops of Hoechst 33342 (NucBlue) stain was then added to each well and incubated for 20 minutes. A final PBS wash was performed, and stained cells were stored in PBS at 4 °C in the dark until microscopy.

2. Method S2 Assessing the recovery rates and WBC depletion rates for MetaCell by immunostaining

Once cultured cell lines reached 75–80% confluency, they were washed with 5 mL of phosphate buffered saline (PBS) and then trypsinised with 5 mL of 0.25% trypsin in EDTA. Trypsin was neutralised via addition of an equal volume of DMEM-FBS, and the cells were then pelleted by centrifugation at 350 *g* for five minutes at 21 °C. The supernatant was discarded, and the cell pellet was resuspended in 20 mL of fresh DMEM + 10% FBS. 100 μL of cell suspension was mixed with 100 μL of trypan blue (Gibco, Cat#15250-061), and the cells were counted on a hemocytometer. Two or three serial factor-of-ten dilutions of the original cell suspension were performed to achieve more dilute working cell suspensions. Cell aliquots of 10, 100, 500, and 10,000 cells were obtained by pipetting appropriate volumes of these cell suspensions into falcon tubes containing a small amount of fresh media. Five 8 mL peripheral venous blood samples were collected from one healthy volunteer in 10 mL potassium EDTA tubes (BD vacutainer lavender 10 mL, Capes Medical, Cat#367525). The peripheral venous blood was then transferred into five 50 mL falcon tubes. To four separate tubes containing peripheral venous blood, 10, 100, 500, and 10,000 CRC cells were added; a fifth, with no cells added, served as a negative control. 1× RBC lysis buffer was added to 8 mL of peripheral venous blood from a healthy volunteer (1 mL of blood–5 mL of buffer) containing the spiked CRC cells, and this mixture was incubated at room temperature for 15 minutes and then filtered through a MetaCell kit, in a filtration process which took around two minutes. Once all blood had filtered through the membrane, the MetaCell membrane was detached and incubated for 8 hours in a six-well culture plate with 4 mL of Roswell Park Memorial Institute Medium (RPMI 1640, Thermofisher Scientific, Cat#11875093) + 10% FBS at 37 °C. Subsequently, 10, 100, 500, and 10,000 CRC cells were added directly to the other

wells of the six-well plate to serve as the positive control and similarly incubated with 4 mL of RPMI + 10% FBS. After eight hours of incubation, the media were removed from all the wells, and the cells were washed with 4 mL of 1x DPBS. The immunostaining procedure was immediately carried out to characterise the CTCs (Methods S1).

The determination of recovery rates for MetaCell for the different cell numbers by immunostaining involved staining the MetaCell membrane with Hoechst 33342 (DAPI), cytokeratins (C11 and AE1/AE3, both conjugated to Alexa fluor 488; AF488), EpCAM- phycoerythrin (PE), CD45 and CD16 (Alexa fluor 647; AF647). The cells were visualised and imaged on a Nikon A1+ inverted confocal microscope at magnification of 200×. The cells that stained positively for Hoechst 33342, pan-cytokeratins, and/or EpCAM and negatively for both WBC markers (CD45 and CD16) were manually counted (except for the group containing 10,000 cells which was used for qualitative assessment of the ability of MetaCell to retain CRC cells) for the unfiltered and the MetaCell-filtered groups. The recovery rates were calculated by:

$$\text{Recovery rate} = M_F / U_F * 100 \tag{1}$$

where
M_F = number of cells manually counted in the MetaCell-filtered group
U_F = number of cells manually counted in the unfiltered group

To assess the WBC depletion rates of MetaCell, two 8 mL peripheral venous blood samples from a healthy volunteer were collected in 10 mL potassium EDTA tubes. From one of the tubes, peripheral blood mononuclear cells (PBMCs) were extracted using the Lymphoprep (MediRay, Cat# N1114544) solution. Into a 50 mL falcon tube, 8 mL of the peripheral venous blood was transferred and diluted with 8 mL of Hank’s balanced salts solution (1:1 ratio). The above diluted peripheral venous blood was mixed gently and then slowly layered on top of 15 mL of Lymphoprep solution. This was followed by centrifugation at 900 *g* at room temperature for 30 minutes. The acceleration was set to 6 and the brake was set to 0. The PBMCs were recovered from the interface with a sterile Pasteur pipette and transferred to a new 50 mL Falcon tube. To the recovered PBMCs from the above step, 50 mL of cold DMEM + 10% FBS was added and spun at 600 *g*, 4 °C for 6 min. The acceleration and brake were set to 9. The cells were resuspended by flicking and addition of 50 mL of Hank's balanced salts solution (Sigma-Aldrich, Cat#H2387-10x1L). If Hank's balanced salt solution was not available, DMEM + 10% FBS could be used. This cell suspension was centrifuged at 300 *g*, 4 °C for 6 min with acceleration and braking set to 9. The supernatant was discarded, and the cell pellet was dissolved in 1 mL of DMEM + 10% FBS and seeded into one well of a 6-well plate. A further 3 mL of RPMI + 10% FBS or DMEM + 10% FBS was added to the well.

The second blood sample was mixed with a RBC lysis buffer in the ratio of 1:5 and incubated for 10 minutes at room temperature. Subsequently, this blood was filtered through MetaCell. The membrane was detached and placed in a different well of the 6-well plate containing the unfiltered PBMCs. The unfiltered and the filtered groups were incubated at 37 °C, 5% CO_2, and 95% humidity for 8 hours in DMEM + 10% FBS. Both samples were stained with fluorescent markers for CD45 and CD16 (Methods S1). Images of the stained cells were captured for both samples on a Nikon A1+ inverted confocal microscope at a magnification of 200×, and mean intensities were calculated for each of the groups using ImageJ (FIJI). The composite image consisting of four channels (DAPI, Cytokeratins-Alexa Fluor 488, EpCAM-PE, and CD45 and CD16-Alexa fluor 647) was split into individual channels by selecting *Image >> colour>> split channels*. The image in the Alexa fluor 647 channel was chosen and converted to an 8-bit image by choosing *Image >> Type >> 8 bit*. The threshold for the image captured in the Alexa fluor 647 channel was set using the auto threshold option by selecting *Image >> adjust >> auto threshold*. Once the threshold was adjusted, the parameters such as area and shape descriptors were selected by choosing *analyze option >> set measurements.* The area covered by the Alexa Fluor 647 was estimated by choosing the *analyze option >> analyze particles* and entering the threshold value determined, to aid in selecting only those cells which fell in that threshold for calculating the total area and the values for the other parameters chosen. These values were then recorded. The mean intensities of the Alexa fluor 647 in unfiltered and MetaCell-filtered blood was calculated using the formula:

$$\text{Mean intensity of Alexa Fluor 647} = \text{Total Area} * \text{\% Area covered by the fluorophore} \tag{2}$$

The mean intensities of Alexa Fluor 647 from both groups was used for calculating WBC depletion rate.

$$R_M = W_F / W_U * 100 \tag{3}$$

where
R_M = % of WBCs remaining in the MetaCell-filtered fraction
W_F = mean intensity of WBC markers (Alexa Fluor 647) in MetaCell-filtered blood
W_U = mean intensity of WBC markers (Alexa Fluor 647) in unfiltered blood
WBC depletion rate = 100 - R_M

3. Method S3 Gene expression analysis for calculating recovery rates and WBC depletion rates for MetaCell

The cells from the MetaCell-filtered and unfiltered groups were lysed in an RLT lysis buffer, transferred to the column provided in an RNA mini easy kit (Qiagen), and centrifuged for 30 seconds at 8000 *g*. An equal amount of 70% ethanol was added to the eluent and mixed well before being transferred to the RNA spin column and centrifuged for 15 seconds at 8000 *g*. The eluent was discarded, and 700 µL of RW1 buffer was added to the RNA spin column and centrifuged for 15 seconds at 8000 *g*. The column was then washed twice with 500 µL of RPE buffer; once at 8000 *g* for 15 seconds and then at 8000 *g* for two minutes. A dry spin was then performed at maximum speed for 3 minutes. Then, 20 µL of RNAse-free water was added to the column, and RNA was eluted and collected in a fresh 1.5 mL Eppendorf tube via centrifugation at 8000 *g* for one minute. The quality of RNA was assessed using an Implen Nanophotometer N60/N50 and further quantified with a Qubit fluorometer (Invitrogen) using a High-Sensitivity RNA assay. RNA was converted to cDNA using a High-Capacity cDNA reverse transcriptase kit. For qPCR, 1 ng of cDNA was used to measure the expression of the CTC markers (*EpCAM*, cytokeratin20). The ct values obtained for the CTC markers were normalised with two housekeeping genes (*RPLP0* and *PPIA*) (Table S4), and the fold change in gene expression was calculated using the $2^{-\Delta\Delta Ct}$ method. Recovery rates were calculated using the formula:

$$\text{Recovery Rate} = M_F / U_F * 100 \quad (4)$$

where

M_F = Log_2 fold expression change of CTC markers in the MetaCell-filtered group

U_F = Log_2 fold expression change of CTC markers in the unfiltered group

To determine the WBC depletion rate, the cells from the Metacell-filtered and unfiltered blood used for the immunostaining analysis were lysed in an RLT lysis buffer, transferred to the column provided in an RNA mini easy kit (Qiagen), and centrifuged for 30 seconds at 8000 *g*. An equal amount of 70% ethanol was added to the eluent and mixed well before being transferred to the RNA spin column and centrifuged for 15 seconds at 8000 *g*. The eluent was discarded, and 700 µL of RW1 buffer was added to the RNA spin column and centrifuged for 15 seconds at 8000 *g*. The column was then washed twice with 500 µL of RPE buffer; once at 8000 *g* for 15 seconds and then at 8000 *g* for two minutes. A dry spin was then performed at maximum speed for 3 minutes. Then, 20 µL of RNAse free water was added to the column, and RNA was eluted and collected in a fresh 1.5mL Eppendorf tube via centrifugation at 8000 *g* for one minute. The quality of RNA was assessed using an Implen Nanophotometer N60/N50 and further quantified with a Qubit fluorometer (Invitrogen) using a High-Sensitivity RNA assay. RNA was converted to cDNA using a High-Capacity cDNA reverse transcriptase kit. For qPCR, 1 ng of cDNA was used to measure the expression of the WBC markers; (Table S4*)*. The expression of *CD45* and *CD16* genes was normalised with two housekeeping genes (*RPLP0* and *PPIA*), and the fold change in gene expression of *CD45* and *CD16* was calculated using the $2^{-\Delta\Delta Ct}$ method for the unfiltered and MetaCell-filtered blood. The gene expression values calculated for both these groups was used for calculating the WBC depletion rates. WBC depletion rates were calculated using the formula:

$$R_M = M_F / UF * 100 \quad (5)$$

where

R_M = %WBCs remaining in the MetaCell-filtered fraction

M_F = Log_2 fold change in gene expression of *CD45* and *CD16* in the MetaCell-filtered group

U_F = Log_2 fold change in gene expression of *CD45* and *CD16* counted in the unfiltered group

This gave the percentage of WBCs remaining in the MetaCell-filtered fraction. The extent of WBC depletion achieved by MetaCell could be calculated by:

$$\text{WBC depletion rate} = 100 - R_M \quad (6)$$
